# Carotid artery thickness in obese patients with chronic renal failure

**DOI:** 10.1590/2175-8239-JBN-2018-0172

**Published:** 2018-10-18

**Authors:** Vitorino Modesto dos Santos

**Affiliations:** Universidade Católica de Brasília, Hospital da Forças Armadas, Brasília, DF, Brasil.

A recent study about the carotid intima-media thickness (CIMT) assessment in obese patients with chronic renal failure (CRF) was published by El-Masrya SA et al.^1^ The authors evaluated 118 Egyptian patients with 30-60 years of age categorized into three groups: I) 45 obese with chronic renal failure (CRF); II) 39 non-obese with CRF; and III) 34 obese without CRF as controls. Major differences related to body mass index (BMI) and lipid profile were described among groups. The group obese without CRF had the highest BMI, waist circumference, total cholesterol, and low-density lipoprotein (LDL) cholesterol. The ultrasound measurements of CIMT revealed that the results were greater than the normal range in all groups, particularly in the group obese without CRF (I: 0.11 ± 0.03 mm; II: 0.09 ± 0.02 mm; and III: 0.172 ± 0.28 mm). They also reported a high correlation between CIMT and waist circumference, but the correlations of CIMT measurement with BMI and lipid profile were unremarkable.^1^ The authors concluded that increased CIMT was more related to obesity than to CRF, and the relationship was more significant with central obesity than with lipid profile, which was emphasized by the authors.^1^ Although parathyroid hormone (PTH) was not assessed in that study, one may compare those data with findings from another study about CIMT in patients with CRF and secondary hyperparathyroidism.^2^

Costa AF et al. reviewed 14 patients aged between 18 and 65 years, categorized into two groups: I) seven with PTH < 200 pg/mL; and II) seven with PTH > 500 pg/mL.^2^ Ultrasound evaluation showed plaques of carotid calcification in 42.86% of patients in group I (lower levels of PTH) and in 71.43% of patients in group II. There was no significant correlation between PTH and CIMT (I: 0.8 ± 0.2 and II: 0.9 ± 0.1 mm). Importantly, there were significant differences in age (p = 0.04) and LDL cholesterol levels (p = 0.03) of patients with and without thickened carotid wall by calcified plaques. There was no significant difference between groups in relation to the time on dialysis, which can have a remarkable effect on the thickness of the carotid arteries.^2^

The Egyptian and Brazilian authors emphasize the role of CRF in cardiovascular mortality, which can be up to 20 times higher than in the general population.^1^^,^ 2 Based on the data herein presented, further prospective studies should be done involving greater number of patients and broader panel of laboratory determinations.
